# Financing malaria

**DOI:** 10.1371/journal.pgph.0000609

**Published:** 2022-06-09

**Authors:** Ravindra P. Rannan-Eliya

**Affiliations:** Institute for Health Policy, Colombo, Sri Lanka; University of Ghana College of Health Sciences, GHANA

## Abstract

The faltering of progress towards malaria elimination follows a plateauing in international financing since 2010. Despite calls for increased international financing, this will be hard to achieve. Both developed country donors and developing countries with malaria face severe fiscal constraints in expanding malaria funding in the next few years. Simply exhorting countries to spend more is unlikely to be successful, just as the Abuja declaration was not, and the developing countries with most malaria burden suffer from weaker economic growth and less capacity to increase domestic financing. One major prospect for substantial new financing is China, but this may depend on established funders yielding influence in the global financing architecture to China and other emerging economies. This argues for greater emphasis on spending available financing better, but improving the impact of international funding is not straightforward. It is associated with significant transaction costs for recipients, impairs the ability of the WHO to coordinate global efforts, and may pressure recipient countries to focus more on commodities and easy wins instead of investing in health systems and management capacity. While more should be done to mitigate these perverse effects, much of this is the unavoidable price of such generosity and the inevitable need for accountability to funders. Ultimately, countries must do more with their own spending, which is often under-counted, but usually far exceeds the international contribution. The experience of Sri Lanka, El Salvador, and China—three countries that eliminated malaria—provides two pointers. First, achieving early and widespread treatment of most malaria cases, which is not the case in much of high burden Africa, may be critical to sustain accelerated elimination. Second, such coverage requires health systems that prioritize access for all services and conditions. Public opinion surveys indicate that this is consistent with what much of the affected population wants, prioritizes, and is willing to finance through higher taxes, which points to weaknesses in accountability of policy to people. International funders could do better to heed what affected populations want and let local partners be responsive to their own public’s preferences.

## Introduction

The global push to accelerate progress on malaria control was made possible by a substantial increase in international financing. New financing mechanisms–notably the Global Fund to Fight AIDS, Tuberculosis and Malaria (GFATM) and the US President’s Malaria Initiative (PMI)–played key roles. The recent slow-down in progress towards malaria elimination, including increases in incidence and mortality rates since 2019 [1], has been accompanied by a plateauing of international financing, leading naturally to concerns that financing may be a constraint to sustaining or accelerating progress. At the same time, there are concerns about the impact of the global financing architecture on the effectiveness of malaria control at the country level. Both sets of concerns have some basis, but the implications for action are not straightforward and require consideration of issues such as accountability and voice. This commentary provides the perspective of a health systems financing expert on these issues and dilemmas, informed by a review of the literature, interviews with malaria experts and officials at global and national levels, a series of reflective discussions with other members of the Working Group on Malaria Governance and stakeholders and participants in the Rethinking Malaria project Advisory Committee, and the personal insights arising from working both at the global level and in a country that only recently eliminated malaria after a century long struggle.

## International financing: Trends and prospects

The push to reinvigorate malaria control has focused on expanding the use of established and new preventive, diagnostic, and treatment interventions. Advocacy was successful in changing GFATM’s originally proposed remit to expand and include malaria, and later in raising new US financing for these interventions, channeled through PMI. These and other similar initiatives resulted in an exceptionally large increase ($2 billion) in international financing from less than $1 billion in the early 2000s to almost $3 billion per year by 2010, mostly provided by the USA, UK, and France, but funding has been static since then [1].

The lack of increase in international financing in recent years is not a failure. The ramp-up in spending reflected the success of the malaria community in capturing the imagination of governments long enough to create new institutional commitments that could sustain funding after their initial enthusiasm had inevitably passed. In the current global environment, however, there is little realistic likelihood of something similar happening soon.

First, high-income countries and other global funders are likely to remain focused on COVID-19 through 2024 and will have little capacity to increase official development assistance (ODA). The pandemic has done huge damage to the balance sheets of leading economies, with most nations increasing their public debt substantially to sustain economic activity. As the world recovers from the pandemic, governments must increase taxes and constrain spending to pay down this debt, making it politically harder to increase ODA spending. Evidence of this can be seen in the UK government’s proposal to reduce its ODA spending to 0.5% of national income, ditching a statutory obligation to keep it at 0.7% of national income.

Second, the recent fashion of using global health security [2–4] as the rationale to finance malaria is unlikely to be helpful. Although a case could be made that ODA for infectious disease control could pay for itself in terms of economic returns for high income countries, such economic cost-benefit arguments are rarely persuasive, as demonstrated by the difficulties in persuading high income economies to provide greater support for global COVID-19 vaccination despite ample evidence of very high economic returns from doing so [5]. The case for malaria is even weaker given that high income nations face little direct risk of malaria, and the limited integration of high burden countries in Africa into global production chains. The fact that malaria affects mostly people in developing countries not only argues against using a health security rationale, which favors the interests of people in rich countries, but is also an argument for using the concept of human security instead, since the human security approach puts the stress on the security, welfare and self-identified needs of the populations most affected by malaria [6].

The one major opportunity for diversifying and increasing international malaria funding is probably China, whose ODA footprint will grow as it emerges as the largest economy in the next decade. Although China is currently a minor player in the malaria funding space, malaria has been a top priority in its health ODA to Africa [7, 8], the country brings its own recent experience of achieving malaria elimination [9], and the country is a major producer of diagnostic and therapeutic interventions. Malaria elimination in Asia and Africa also aligns well with China’s Belt and Road Initiative, a key part of its ODA strategy.

However, two barriers constrain China’s potential contribution. The first is that China’s ODA program is very much a work in progress [10]. Its official ODA agency, CIDCA, was only established in 2018 and remains small, making effective engagement difficult from both sides. China also lacks a deep ecosystem of contractors, research institutions and thinktanks, NGOs and even development experts that would help inform and implement a deep engagement with the global malaria community as well as effectively translate its own experience. This will not change rapidly, but it suggests a role for enlightened funders, development partners and academic institutions to engage in building and mentoring China’s capacity to do so. The second barrier is one of voice. The perception that many multilateral entities remain dominated by Western nations and do not provide adequate representation to emerging economies like China is real and affects multilateral institutions ranging from the IMF and World Bank to COVAX [11], and it may be an issue for GFATM and RBM. Effective inclusion of China and also the perspectives of other emerging non-Western powers will require decolonization of these institutions too [12], which will be challenging for stakeholders opposed to engagement and yielding or sharing influence [13], and likely even harder as Western countries adopt a more confrontational posture to China.

## Domestic financing: Trends and prospects

Despite the focus on international financing, the reality is that domestic financing for malaria, from government and private sources, has always been far greater. The financial contribution of developing countries is systematically under-counted because most efforts to track malaria financing only consider programmatic spending by malaria control programs, and do not consider and count the much larger spending by general health services in the routine treatment of malaria and suspected malaria cases, which also includes private expenditures by households. Even when treatment and routine care of malaria is counted, this is often restricted to the costs of consumables and direct program staff and does not consider the wider health service support and infrastructural costs. This stems from the difficulty of reliably assessing and allocating total health service spending by disease, which even OECD economies have difficulty doing on a regular basis. A detailed 2015 study found that 5–10% of all inpatient and outpatient episodes in the Solomon Islands were due to malaria and that malaria cost more than the average treatment episode [14], implying that 7–15% of the country’s routine medical spending was for the management of malaria, substantially greater than the spending reported by the malaria control program [15]. Similarly, many small-scale studies from Nigeria [16, 17], a high burden country in West Africa, suggest that 10–40% of outpatient and inpatient episodes in the country are due to malaria and that malaria treatment accounts for a significant share of household budgets, making malaria probably the leading cause of healthcare spending in the country.

Since most malaria spending is by the affected countries themselves, and since many developing economies have been growing faster than developed economies in recent years, some have suggested that the burden of increased financing could be shifted more to the affected countries themselves. However, this ignores a growing mismatch between where the remaining malaria burden is and the economic capacity of countries. Malaria decline has been greatest in the developing regions with highest economic growth, principally South-East and South Asia, leaving the bulk of the malaria challenge in Africa where countries are least able to leverage domestic financing owing to lower incomes, lower rates of economic growth and less fiscal capacity. Although these countries can theoretically mobilize greater financing through increased taxation and increased allocation of government budgets to health (including malaria), it is instructive to consider the impact of the 2001 Abuja Declaration, when African Union governments committed to allocate 15% of government budgets to health. In the subsequent two decades, very few African governments have met its target (only two in 2018), and by 2015 most had reduced allocations [18]. What this tells us is that official commitments or exhortations to increase spending are unlikely to work in the more constrained economic environment that developing countries likely face in coming years, as they simultaneously recover from the COVID-19 pandemic and struggle with the commodity supply and inflation shocks arising from conflict in Europe. They will need a more persuasive political economy rationale. That will probably not arise from arguments that controlling malaria improves health and economic productivity, which are valid, but are clearly insufficient.

These constraints on increased domestic and international funding for malaria should lead us to be realistic about prospects for increased financing in the next few years. Although these cannot be completely discounted, we should give more attention to what can be done if funding does not increase. Here the answer is obvious—we need to do more with what we have or even more with less. From a financing perspective, the focus should be on increasing the efficiency and effectiveness of financing—both domestic and international—in controlling and reducing malaria transmission, and especially in the high burden countries, most of which are in Africa [1].

## Does international financing and the global financing architecture impair progress?

Although international funding is the smaller part of overall malaria financing, it is still important, because of how it influences malaria control policy and the structure and organization of national malaria control programs, and its role in the financing of key commodities in many countries. If malaria financing is to be made more effective, is there potential for improving the impact of international funding? Here, there are at least four sets of issues.

The first is that international financing of malaria imposes significant transaction costs on recipient countries, a form of inefficiency that reduces the value of each donor dollar, although donor-funded global pooling of commodity purchases partly offsets this. One part of this stems from the existence of several substantial funders, *i*.*e*., GFATM, PMI, BMGF (Bill & Melinda Gates Foundation) and other bilateral ODA agencies, which fragments the funding flows to and within countries, making it more difficult and burdensome for national programs to coordinate and manage funding and control activities. Key informants at country level frequently report the problems they face in managing multiple funders, as well as the asymmetry in power relations that arises that make it more difficult to effectively manage international funders.

These transaction costs also arise from the skewed incentives that international funding can create, either for governments to favor some activities over others, or the incentives created for individuals, for example when local officials or experts are paid more to work for donor funded programs. These problems are real and significant, but they are not unique or specific to malaria, affecting the whole range of ODA-funded activities, although more of an issue in the relatively well-funded and popular health sector than in others.

The transaction costs that ODA imposes on countries have been acknowledged for at least two decades and have led to several efforts to streamline ODA flows to countries, as well as to reduce the burden and perverse incentives within countries. The 2005 Paris Declaration on Aid Effectiveness, for example, called on countries to ensure that donor efforts complement each other, and for donors to concentrate their aid and expertise where it can bring the biggest benefits. Whilst all major funders have signed up to the Paris Declaration and made various commitments to pursue best practices, more can always be done. Within the malaria space, a forum to discuss these problems and for key funders to do more to find ways of improving practices, learning from what we know can work, could be helpful. This may well be an area that the World Bank or the WHO could lead on given the obvious benefits.

Realistically, however, ODA financing will always be associated with its own transaction costs. So, countries must decide for themselves whether the net benefits of taking ODA funding outweigh the transaction costs, and if they can do more, learning from other countries, to better manage the terms of their interactions with external malaria funders. The latter might mean, for example, being more assertive that donors should divide their support by type of intervention as opposed to by subnational region (which appears to be particularly problematic), and that donors provide more core support to national program management and coordination activities. Here again, support by development partners for learning about lessons in strengthening country management and for dissemination of best practices to country counterparts could help.

The second set of issues related to international financing concerns the governance of the global funding architecture and perceptions that current arrangements give too much influence to some actors, particularly GFATM and the United States, and too little to others, specifically the WHO. The WHO concerns are understandable. It is rightly the lead agency for directing the global malaria control effort, but its chronic lack of core funding means that it has less capacity to steer policy and coordinate actual implementation. Instead, the bulk of international financing flows through GFATM. And changes at RBM, which include shifting its Geneva offices out of WHO headquarters, have reduced the voice that the WHO has in its governance and the closeness of their day-to-day relationship. However, it is unclear how these concerns can be completely resolved, given that the substantial funding that high-income country governments give to GFATM for malaria probably would not flow to the WHO or even to malaria control if GFATM didn’t exist. The trade-off involved in securing additional funding commitments for malaria is that the relevant funders expect more accountability and influence, which the WHO cannot provide given that its constitutional basis favors democratic accountability to all members of the global community and not simply to countries or donors with greater financial resources.

The contrasting emphasis on accountability to funders not only works at the level of GFATM decision-making, but also goes through to the accountability that GFATM imposes on beneficiary countries when spending money. Although this generates criticism around the constraints and burden it creates for countries, the increased accountability that countries have faced when spending money has almost certainly been positive and helped accelerate and keep on track funded malaria control activities. It should also be admitted that the level of accountability and pressure that GFATM has introduced is not something that the WHO could have done well. Given the inherent trade-offs between more funding and accountability and influence, the best option would be for major funders to provide more direct funding to the WHO to strengthen its core steering, coordination, and country technical support functions, and for RBM to strengthen its relationship with the WHO.

The third set of issues related to international financing concerns whether the global financing architecture impairs the malaria elimination effort by shaping or altering what is done. This is different from the problem of transaction costs associated with international financing, which may increase the effective price of activities [a form of technical inefficiency] but does not alter the mix of interventions [a matter of allocative efficiency]. One way this could happen is if global financing results in suboptimal allocation of investments across different interventions. In theory, this should not happen since all major funders aim in principle to support and align with the WHO global malaria strategy and country-developed national strategic plans with their mix of targets and intervention approaches. However, in practice this may happen in three ways.

First, despite the consensus that strong program management and surveillance should be priorities for investment [19], these, especially management strengthening, have not been prioritized in international funding. This may be because strengthening management is seen as too hard or lacking effective solutions: donors frequently shy away from the arduous process of strengthening institutional capacity in favor of quick fixes to get their immediate objectives done. Additionally, donors may sometimes prefer weak country partners as this makes it easier for them to set the agenda and to influence how national plans are implemented. Unfortunately, strengthening management capacity may be critical to achieving more with less, both globally and within national malaria control efforts. Sri Lanka and China are good examples of this, since both their successful malaria elimination efforts were done at low cost and with efficient use of resources by public sector managers. But their experience suggests that the problem of better management might be something that can only tackled effectively by countries themselves taking ownership, since it is intimately related to the issue of local accountability. Nevertheless, international funders could do much more to learn from successes such as Sri Lanka [20], and to support translation and South-South sharing of relevant lessons, as well as supporting long-term efforts to strengthen health management where countries themselves take the lead.

Second, international financing appears to favor commodities. Bilateral funders are ultimately accountable to their own politicians and taxpayers. This likely favors investment in commodities and other actions that can be visibly associated with impact and are easily accounted for. This is quite apparent when perusing the annual report by PMI to the US Congress, which focuses on such concrete indicators as bed nets and diagnostic tests distributed and child deaths prevented, as opposed to improvements in surveillance and management systems [21]. In a normative sense, this is not wrong—indeed a key requisite for successful development should be to ensure that governments are accountable to their people for what they do with the people’s money [22]; but this excessive reliance on international funding can distort the accountability away from the people who suffer malaria and who often have weak accountability over their own states to people in faraway lands who do not, with potentially negative impacts on malaria control.

Finally, a frequently expressed concern is that international financing undermines local ownership of malaria control. There is some truth to this, but it also depends on country motivation. Where countries are strongly committed to the goal, it is much less likely that international financing will damage or weaken local control. The real focus should be on how to ensure that countries have strong ownership that is resilient to the impact of international financing.

## What could we do differently?

To accelerate malaria elimination progress in a scenario where increased international funding is unlikely, the critical question is: What could we do differently?

The “Rethinking Malaria Strategy in the Context of COVID–19” project is based on the premise that business as usual is not enough. It is beyond the scope of this paper to provide a proper answer to this question, but I will offer some thoughts that link to points raised in the preceding discussion about malaria financing and seek lessons from three national success stories. This draws on the experiences of Sri Lanka, China, and El Salvador [23]—three countries that succeeded in eliminating malaria at relatively low cost—whilst contrasting them with one high-burden African country, Nigeria.

Whilst acknowledging the differences in the epidemiology of malaria between regions, the most striking difference between these countries and the situation in much of Africa today is the role that their general health services and treatment played in controlling malaria. In all three countries, the population’s routine use of all medical services was much more frequent and appears to have been associated with a much higher fraction of malaria or fever cases being seen by providers, usually earlier, and being treated. The Sri Lankan situation is clear. From the 1950s, rates of medical care use in Sri Lanka were exceptionally high, increasing from 2 to 7 doctor consultations per capita per year by the 2010s, when malaria was finally eliminated. This high rate of use of medical treatment included fever cases [24], meaning that the treatment of malaria played a significant role in the control of malaria transmission. China’s experience is similar, with its unique emphasis on mass drug administration, and in the later stages like Sri Lanka on detection and treatment of all cases [25, 26], whilst elimination in El Salvador was characterized by an aggressive treatment policy in which 95% of people receiving treatment did not have malaria with less emphasis on vector control and bed-nets [27]. This can be contrasted with the situation in sub-Saharan Africa where most health systems are weak, and many or most malaria cases never receive treatment [28]. Estimates suggest that in 2015 only 20% of symptomatic RDT-positive children under 5 years old in Africa received an ACT, with less than 40% taking any antimalarials in Nigeria [29]. Such differences in treatment coverage matter because recent research shows that early treatment of malaria cases, even if not always effective, can substantially reduce onward transmission in Africa [30], and is critical globally.

In short, a key challenge in eliminating malaria in many high burden countries is the weakness and low coverage of the overall health system and local health services, within which malaria control is embedded [4]. That weakness translates in too few malaria cases being treated early or at all, which makes it more difficult for other control interventions to reduce transmission sufficiently to achieve rapid control. Further, such weaknesses will matter more when transmission begins to fall.

This raises the question as to how Sri Lanka got people with malaria or fever to seek medical care so frequently. The answer lies not in what the malaria control program did, but in overall health policy. Since the 1930s, Sri Lanka pursued a strategy that prioritized universal access to medical care regardless of disease, which meant abolishing user fees, building as many healthcare facilities as possible to maximize geographical access, and empowering and training managers, who were always doctors, to constantly do more with less to stretch the limited public budget [20, 31]. Sri Lanka did not do these things because of advice from international funders (they often advised the opposite! [32]), but because its political economy—especially the election of all governments by universal suffrage from 1931—made its governments highly responsive to ordinary people. And critically although the initial expansion of healthcare in Sri Lanka was driven by the devastating impact of malaria on rural households, the people weren’t so much interested in the experts’ concern with better malaria prevention, as in having immediate access on demand to a doctor or medicines when sick or a hospital bed when they needed nursing [31]. Indeed, although political pressures arising from malaria were critical in the expansion of Sri Lanka’s health system from the late-1930s, once overall healthcare access was achieved, efforts to use malaria control as an issue of political mobilization were ineffective by the 1970s [33].

Economic analysis since the 1990s has shown that the approach that Sri Lanka took is key to increasing use of medical care in other developing countries, especially removing price barriers and minimizing physical distance to facilities [34]. Such policies are likely to be highly popular with people in many high-burden countries. For example, in Nigeria, where most people have low confidence in their health system, health was the most important issue for voters in 2019, and when asked which health promises were most attractive, 53% cited free or cheaper healthcare, followed by 11% for more healthcare facilities, with only 8% mentioning better malaria control [35]. Other data from Nigeria also show that simple improvements in local healthcare services can substantially increase support for politicians [36], whilst people will support higher taxes to pay for public services, with support greater for increasing taxes on the rich [37]. This last data point is intriguing as it indicates that a better approach to increasing health spending in Africa would be to frame it as an issue of domestic political self-interest instead of as an international obligation and demonstration of good behavior.

This raises the question as to why such public preferences in African countries, which would facilitate faster malaria elimination through strengthening health systems (and support raising taxes to pay for health), have not had the same impact as in Sri Lanka. Here I can only speculate. Perhaps one reason why politicians have not done what is likely to be popular with the public and so presumably in their own interests has been the relative weakness (until recently) of electoral politics in these countries, information failures in the political market, plus the greater influence of external funders in setting health (and malaria) policies and their frequent failure to ground policy advice in terms of political rationality. Another reason may be that it takes time for politicians to take initial actions to improve services, for voters to then realize their vote counts, and for a positive dynamic between politicians and voters drives further improvement in health services. This was certainly the case in Sri Lanka [31].

## Making financing work better requires strengthening country accountability

My overall assessment about international financing for malaria is that it is not realistic to expect an increase. The priority should be on how to make existing international financing flows and domestic financing work better together in achieving faster and effective malaria control.

In health financing, the saying “he who pays the piper calls the tune” is often true. But it can distract us from considering issues related to institutions and the effectiveness of accountability and voice. It is quite likely that the people living in high-burden countries already finance the bulk of malaria spending, far more than the one third reported by the WHO and others [15]. However, in high burden countries local public financing frequently does not reflect the priorities of the people, particularly in the provision of universal access to healthcare. Governments mobilize insufficient taxes to pay for health, they spend too little to provide adequate services that people want, and they fail to invest in and incentivize health managers to use limited resources well. Unless these broader issues are addressed by the malaria endemic nations themselves, it may be hard to accelerate or sustain malaria elimination in the highest burden countries.

The malaria community cannot by itself solve these wider health system challenges that constrain malaria elimination. And they may well be more critical in high-burden countries with high transmission where no single subset of interventions can be sufficient. To the extent that they are fundamentally local problems of accountability and government performance, they also cannot be solved by international funders. However, funders could do more to do no harm and to align with other efforts to improve overall healthcare access. They could also make serious investment in building local capacity to manage health services. But ultimately, they need to pay more attention to what people in high burden countries want rather than what people and experts in high income countries expect, and cede more agency to those peoples and their governments.

## Supporting information

S1 File "Rethinking Malaria in the Context of COVID–19," a global engagement organized by Harvard University. (DOCX)
