# Supplementary material for: Financing malaria
Source: PLOS Glob Public Health. 2022 Jun 9;2(6):e0000609. doi: 10.1371/journal.pgph.0000609 (PMC10021190; doi:10.1371/journal.pgph.0000609)
Supplement: S1 File — (DOCX) [file pgph.0000609.s001.docx]

**S1: "Rethinking Malaria in the Context of COVID–19," a global engagement organized by Harvard University.**

Harvard University [Internet]. Boston: Rethinking Malaria/COVID19; c2021 [cited 2021 Sept 1]. Defeating Malaria: From the Genes to the Globe Initiative; [about 3 screens]. Available from <https://www.defeatingmalaria.harvard.edu/rethinking-malaria/>.
